# Postpartum Diet and the Lifestyle of Korean and Chinese Women: A Comparative Study

**DOI:** 10.3389/fpubh.2022.803503

**Published:** 2022-04-06

**Authors:** Jia Li, Heewon L. Gray, Sohyun Kim, Haeryun Park, Youngmi Lee, Hongmie Lee, Kyunghee Song

**Affiliations:** ^1^Department of Nutrition, Jinhua People's Hospital, Jinhua, China; ^2^Department of Food and Nutrition, Myongji University, Seoul, South Korea; ^3^Department of Community and Family Health, University of South Florida, Tampa, FL, United States; ^4^Department of Food Science and Nutrition, Daejin University, Pocheon, South Korea; ^5^Graduate School of Education, Nutrition Education Major, Daejin University, Pocheon, South Korea

**Keywords:** dietary behavior, health-related behavior, nutrition education, psychological health, postpartum Korean and Chinese women

## Abstract

**Objective:**

The study investigated and compared postpartum diet and behaviors, nutrition education, and psychological health status among Korean and Chinese postpartum women (0–6 months after delivery).

**Methods:**

A total of 221 Korean mothers in Gyeonggi-do (South Korea) and 221 mothers in Jinhua (China) participated in the survey between November and December 2018, and the results were statistically analyzed using the SPSS Statistics 25.0 software.

**Results:**

Many postpartum behaviors, such as postpartum diet pattern and care duration differed between Korean and Chinese mothers. The former showed a stronger desire for nutrition education compared with the latter (*p* < 0.001). Korean mothers' life and meal satisfaction, and contentment with their husband's support were all ~0.5 points higher compared with Chinese mothers, particularly regarding spousal support (*p* < 0.001). Postpartum depression stresses positively correlated with postpartum infant care stress and negatively correlated with life satisfaction. In addition, primipara mothers were more in need of infant care support and guidance concerning appropriate baby-feeding techniques compared with multipara mothers.

**Conclusion:**

Traditional culture was a crucial factor that influenced the perceptions of postpartum women in South Korea and China. Culturally tailored nutrition education and exercise programs may benefit Korean and Chinese women after childbirth.

## Introduction

The postpartum period refers to the first 6 weeks after childbirth, also known as the fourth trimester. New mothers may experience a range of physiological and psychological issues after childbirth including hormonal changes, metabolism disorders, postpartum depression (PPD), childcare stress (CS), and anxiety. Although healthcare has significantly improved over the past decades, the data indicated that maternal mortality remains high (1). According to a World Health Organization report, approximately 11–17% of women died during childbirth, and 50–71% of maternity deaths occurred in the postpartum period (2). In addition to medical reasons, psychological health has become a topic of focus among Korean and Chinese researchers not only because it affects mortality during the postpartum period and the future health of mothers but also because how it manifests may vary according to culture.

The most common and serious mental disorder during the postpartum period is PPD, a type of mood disorder that typically begins with childbirth and can predispose women to subsequent depression and other mental and physical health problems (3). Based on a review that collected and analyzed 40 relevant studies from 40 countries, the prevalence rate of PPD ranged from 0.5 to >60% (4).

The exact cause of PPD is unclear but is likely related to a combination of physical and emotional factors. Studies have explored several possible individual factors associated with PPD including medication (5), maternal age, educational level, smoking history, hereditary factors, marriage status, family economic status, social support, and prenatal depression (6, 7). Conversely, postpartum anxiety and stress were a burden on mood and mental health, which correlated with harmful consequences for mothers, children, and families. A study found stress to be significantly linked to pre-pregnancy body mass index and PPD (8).

The relationship between stress and mental health revealed that 30% of mothers reported experiencing psychological and physiological distress due to postpartum stress (9). Moreover, many studies found that the tendency to become anxious or depressed was greater in the presence of several risk factors, such as rapidly changing family structures, role-changing (10), and stressful life events during pregnancy (11), as well as parturition costs (12).

To our knowledge, the factors affecting PPD and stress are complex, especially for the comparison of influencing factors in different countries. Since cultural custom plays an essential role in all human experiences and comprises several traditional beliefs, ideas, perspectives, cognitive styles, and dietary habits and behaviors, increasing attention has been drawn to the aspect of cultural background as it affects the postpartum period (13). For example, in South Korea and China, many new mothers believe that if postpartum care is inadequate, various symptoms of discomfort can arise and have a lifelong effect. Stress and PPD are culture-bound phenomena that are influenced by traditional beliefs (14).

The traditional Chinese word *zuo-yue-zi*, as well as the Korean word *Samchilil* (which means “confinement period” or “doing the month”) (15), refers to an integrated set of customs that include beliefs and practices relevant during the postpartum period (16). In Asia, postpartum women have the custom of confinement, during which they have special diets, special behaviors, etc. Following childbirth, both the woman and her newborn need special postpartum care including help related to the acceptance of role-changing, postpartum activities, the maintenance of body hygiene, and food consumption. These steps are believed to restore maternal health and prevent future diseases. Many health problems, whether physiological or psychological, can be detected early through effective postpartum care that is based on cultural background (17). However, to our knowledge, no study has investigated and compared psychological health status and its relationship to postpartum traditional practices between Korean and Chinese cultures.

To address these issues, we sought to describe the similarities and differences in the confinement behaviors of postpartum South Korean and Chinese women. We investigated the strategies, e.g., culturally tailored nutrition education and exercise programs that may benefit Korean and Chinese women following childbirth.

## Methods

### Setting and Participants

A total of 221 women in South Korea (from the Gyeonggi region) and 221 women in China (from the Jinhua region) were recruited for this research, and the survey was conducted from November 2018 to January 2019. This included responses from 139 women using an online panel provider and responses from 303 women recruited from local hospitals or maternity care centers. Three private maternity care centers in the Yongin and Jinhua regions were selected because the managers of these centers were willing to participate in the study. Each participant was assured of confidentiality and had the right to decline participation or remove themselves from the study at any time. The eligibility criteria for participants were as follows: women (1) in good health, without any known diseases (e.g., hypertension, diabetes, and gestational disease); (2) aged ≥18 years; (3) within the period from childbirth to 6 months postpartum; (4) able to speak and read Korean or Chinese; (5) able to cooperate with data collection methods.

### Ethical Considerations

This study was conducted in accordance with the declaration of Helsinki and approved by the Institutional Review Board of Myongji University (No.: MJU2018-10-002-02).

### Measurements

A series of self-reported questionnaires were administered to assess new mothers' characteristics and postpartum routines. The data collection methods included the following aspects:

(a) A sociodemographic questionnaire was employed to obtain sample characteristics including age, educational level, occupation and current employment status, household income level, number of children, delivery mode, and infant feeding type.

(b) A retrospective questionnaire was administered to analyze and compare diet and hygiene-related behaviors, which comprised special meals, physical activity, and traditional health beliefs from childbirth to 2 months postpartum.

(c) Women's satisfaction with their current life was measured using a 5-point Likert response scale applying the following range: very dissatisfied, (1); somewhat dissatisfied, (2); neither satisfied nor dissatisfied, (3); somewhat satisfied, (4); very satisfied, (5).

In addition, participants were asked to recall information about nutrition education that they had received, such as the experience and frequency of joining relevant courses, sources of information, topics of interest, and type of education lecture. Nutritional knowledge consisted of 15 questions, including nine general nutritional knowledge questions (such as “high nutritional value food is high calorie food”) and six pregnancy nutritional knowledge questions (for example, the main carbohydrate of breast milk production). Each question was answered with “yes” or “no”, and 1 point was given for correct answer and 0 point for incorrect answer. The perfect score of nutritional knowledge was 15 points.

The Edinburgh Postnatal Depression Scale (18) (EPDS, Korean and Chinese versions) and the Childcare Stress Inventory (19) (CSI) were used to measure the psychological status of the participants. The EPDS, developed by Cox et al. (18), is used worldwide as an effective PPD screening scale. The scale indicates the mother's state of mind felt during the preceding 7 days.

Studies evaluated the reliability and validity for the Korean (20) and Chinese (21) versions. In the current study, Cronbach's alpha (α) was 0.80.

The EPDS includes 10 items, such as “I felt sad or miserable” or “The thought of harming myself has occurred to me a couple of times”; each item was rated on a 4-point scale ranging from “no, not at all” (0) to “yes, all the time” (3). The possible depression score was ≥10, with a higher score indicating more significant depression. Mothers who scored >13 were likely to have a depressive illness of fluctuating severity.

The CSI was developed to index stressful postpartum events specifically related to childcare. We chose 15 items that focused on childcare; mothers were asked to rate each event for its stressfulness on a 5-point scale ranging from “absolutely not” (1) to “very much” (5) if they had experienced an event during the first 2 months after childbirth. The score item was rated (1) if they had experienced none.

Accordingly, the possible total score range of this scale was 15–75, with a lower score indicating a lower level of CS. Cronbach's α was 0.82 for this measurement.

### Statistical Analysis

All statistical analyses were performed using the SPSS Statistics 25.0 software program, and the statistical significance was set as p < 0.05. The data were analyzed using descriptive statistics, a *t*-test, a chi-square (X^2^) test, and Duncan's test, as well as Pearson's correlation coefficient. Frequency and percentage were calculated and an X^2^ test was used to describe the characteristics of women concerning demographics, diet, and health behaviors. The results were expressed as mean ± standard deviation (SD) for PPD, CS, and satisfaction. The assessment of significant differences between mean values was performed using a *t*-test. Correlations between postnatal depression and CS, education, and feeding type among mothers were determined using Pearson's correlation coefficient.

## Results

### Characteristics of the Participants

The sociodemographic characteristics of mothers in the two countries are compared in Table 1. Overall, 74.6% (72.9% Korean/76.5% Chinese) of postpartum women's age ranged from 26 to 35 years; 17.2% were older than 36 years, and only 8.1% were younger than 25 years. The results of education level showed that the bulk of participants in both countries had obtained a college degree, which included 177 (80.1%) Korean and 150 (67.9%) Chinese mothers. A significantly higher proportion of women in South Korea were housewife (39.4%) compared with only 14% of Chinese women.

**Table 1:** Demographic and anthropometric indices of participants by country.

**Classification**		**Korea**	**China**	**Total**	**t or X^**2**^**	***p***
		**(*n =* 221)**	**(*n =* 221)**	**(*n =* 442)**		
		**n (%), M ±SD^†^**	**n (%), M ±SD**	**n (%), M ±SD**		
Age (yrs)	≤ 25	13 (5.9)	23 (10.4)	36 (8.1)	14.62^**^	0.002
	26~35	161 (72.9)	169 (76.5)	330 (74.6)		
	≥ 36	47 (21.3)	29 (13.1)	76 (17.2)		
Height (cm)		161.97 ± 4.52	162.13 ± 4.16	162.05 ± 4.34	−0.39^†^	0.965
Weight after childbirth (kg)		60.96 ± 9.30	58.87 ± 6.15	59.92 ± 7.95	2.79^**^^†^	0.006
BMI		21.08 ± 2.85	20.34 ± 1.88	20.71 ± 2.44	3.24^**^^†^	0.001
BMI^‡^	Underweight	41 (18.6)	18 (8.1)	59 (13.3)	17.48^***^^‡^	0.004
	Normal weight	152 (68.8)	177 (80.1)	329 (74.4)		
	Overweight	28 (12.7)	26 (11.8)	54 (12.2)		
Education level	≤ High school	27 (12.3)	49 (22.2)	76 (17.2)	10.63	0.014
	College	177 (80.1)	150 (67.9)	327 (74.0)		
	≥Graduate school	17 (7.7)	22 (10.0)	39 (8.8)		
Occupation	Production worker/laborer	0 (0.0)	9 (4.1)	9 (2.0)	78.56^***^	<0.001
	White-collar workers	134 (60.6)	181 (81.9)	315 (71.3)		
	Housewife	87 (39.4)	31 (14.0)	118 (26.7)		
Monthly household income (10,000 won/yuan)	≤ 100/ ≤ 4,000	0 (0.0)	2 (0.9)	2 (0.5)	112.52^***^	<0.001
	101–700/4,001–16,000	193 (87.3)	134 (60.6)	327 (74.0)		
	≥701/16,001	28 (12.7)	85 (38.5)	113 (25.6)		
Family type	Nuclear family	207 (93.7)	157 (71.0)	364 (82.4)	39.85^***^	<0.001
	Joint family	14 (6.3)	64 (29.0)	78 (17.6)		
Number of children	1	151 (68.3)	132 (59.7)	283 (64.0)	4.14	0.126
	2	66 (29.9)	81 (36.7)	147 (33.3)		
	≥3	4 (1.8)	8 (3.6)	12 (2.7)		
Delivery mode	Natural childbirth	128 (57.9)	127 (57.5)	255 (57.7)	15.18^**^	0.001
	Caesarean section	61 (27.6)	84 (38.0)	145 (32.8)		
	Caesarean section after natural delivery	32 (14.5)	10 (4.5)	42 (9.5)		
Baby feeding type	Exclusive breastfeeding	49 (22.2)	92 (41.6)	141 (31.9)	19.28^**^	<0.001
	Breastfeeding + formula	155 (70.1)	117 (52.9)	272 (61.5)		
	Formula only	17 (7.7)	12 (5.4)	29 (6.6)		

† *M ± SD represents mean ± standard deviation*.

‡ *Underweight: <18.5,normal:18.5-22.9, overweight:≥ 23*.

** *p < 0.01*,

*** *p < 0.001*.

Among white-collar workers (sales, office worker, administrative, professional, etc.), Korean mothers accounted for 60.6%, whereas Chinese mothers represented 81.9%. At the time of conducting this study, the percentages of women who had stopped working because of pregnancy or childbirth were 52.0% and 37.6% compared with the total number of mothers in South Korea and China, respectively. For household income, 27.6% of Korean women's families earned 3,000,000–4,000,000 won monthly, and 38.5% of Chinese women's families earned above ¥16,000 a month. The sample comprised 68.3 and 59.7% primiparous women from South Korea and China, respectively. Most of the women (57.9% Korean/57.5% Chinese) opted for a vaginal birth, which is a natural method of childbirth. According to the results for infant feeding type, 49 (22.2%) Korean and 92 (41.6%) Chinese mothers breastfed exclusively, whereas 70.1% of Korean and 52.9% Chinese mothers adopted a combination of breastfeeding and formula.

### Postpartum Diet and Behaviors

Postpartum diet and behaviors after giving birth are summarized in Table 2. Briefly, women in South Korea and China followed confinement customs but beliefs about this varied, e.g., food and activity principles during the first 2 months postpartum. Concerning the duration of confinement behaviors, a period <7 days was accounted for as “not sitting the month”; 4.5% of the total participants belonged to this group. The custom of “staying at home” for 7–21 days appeared to have been observed by 71% of Korean women compared with 4.1% of Chinese women (p < 0.001). Most of the Chinese women (71.1%) opted for 22–35 days of resting at home rather than leaving their homes.

**Table 2:** Comparison of confinement diet, behaviors after delivery, nutrition information sources and the need for nutrition education among Korean and Chinese postpartum women.

**Classification**		**Korea**	**China**	**Total**	**p**
		**(*n =* 221)**	**(*n =* 221)**	**(*n =* 442)**	
		**n (%), M ±SD^†^**	**n (%), M ±SD^†^**	**n (%),M ±SD^†^**	
First meal after delivery	Brown sugar water	0 (0.0)	9 (4.1)	9 (2.0)	<0.001
	Seaweed soup	140 (63.3)	8 (3.6)	148 (33.5)	
	Egg soup/Steamed egg	0 (0.0)	28 (12.7)	28 (6.3)	
	Noodle	0 (0.0)	52 (23.5)	52 (11.8)	
	Porridge	76 (34.3)	103 (47.6)	179 (40.4)	
	Chicken soup	0 (0.0)	12 (5.4)	12 (2.7)	
	Other	5 (2.3)	9 (4.1)	14 (3.2)	
Confinement period(days)	<7	20 (9.0)	0 (0.0)	20 (4.5)	<0.001
	7~56	194 (87.8)	217 (98.2)	411 (93.0)	
	>56	7 (3.2)	4 (1.8)	11 (2.5)	
Nutritional supplements	Yes	210 (95.0)	165 (74.7)	375 (84.8)	<0.001
	No	11 (5.0)	56 (25.3)	67 (15.2)	
Exercise during confinement period	Yoga	88 (39.8)	157 (71.0)	245 (55.4)	<0.001
	Swim	13 (5.9)	2 (0.9)	15 (3.4)	
	Walk	57 (25.8)	39 (17.6)	96 (21.7)	
	Jog	3 (1.4)	13 (5.9)	16 (3.6)	
	Other	5 (2.3)	5 (2.3)	10 (2.3)	
	No exercise	55 (24.9)	5 (2.3)	60 (13.6)	
Perceived sleeping quality	Excellent	16 (7.2)	44 (19.9)	60 (13.6)	<0.001
	Interrupted sleep 1-2 times	121 (54.8)	126 (57.0)	247 (55.9)	
	Hardly sleep	84 (38.0)	51 (23.1)	135 (30.6)	
Sleep with baby	Yes	144 (65.2)	201 (91.0)	345 (78.1)	<0.001
	No	77 (34.8)	20 (9.0)	97 (21.9)	
Total nutrition knowledge score	12.36 ± 1.58	10.61 ± 1.75	11.49 ± 1.88	<0.001	
Degree of perceived nutrition knowledge	3.00 ± 0.81	2.92 ± 0.83	2.96 ± 0.82	0.326	
Degree of need for nutritional education	4.10 ± 0.79	3.75 ± 0.74	3.93 ± 0.78	<0.001	
Experience of nutrition education (times)	Never	158 (71.5)	44 (19.9)	202 (45.7)	<0.001
	1~2	43 (19.5)	134 (60.6)	177 (40.0)	
	3~4	12 (5.4)	40 (18.1)	52 (11.8)	
	≥ 5	8 (3.6)	3 (1.4)	11 (2.5)	
Pay for online class	Yes	15 (6.8)	20 (9.0)	35 (7.9)	0.378
	No	206 (93.2)	201 (91.0)	407 (92.1)	
Nutrition education approach	Academic lecture	38 (17.2)	14 (6.3)	52 (11.8)	<0.001
	Practice class	93 (42.1)	90 (40.7)	183 (41.4)	
	Group study	16 (7.2)	77 (34.8)	93 (21.0)	
	Personal consult	34 (15.4)	18 (8.1)	52 (11.8)	
	Online class	40 (18.1)	22 (10.0)	62 (14.0)	
Content of nutrition education	Breast-feeding	80 (36.2)	55 (24.9)	135 (30.5)	<0.001
	Complementary feeding	65 (29.4)	38 (17.2)	103 (23.3)	
	Cooking method	33 (14.9)	58 (26.2)	91 (20.6)	
	Diet therapy	11 (5.0)	52 (23.5)	63 (14.3)	
	Diet selection knowledge	32 (14.5)	18 (8.1)	50 (11.3)	

† *M ± SD represents mean ± standard deviation*.

Dietary aspects reflected significant differences between the two groups. Seaweed soup was a popular choice among Korean mothers as the first meal after childbirth, whereas Chinese women chose porridge or noodles. A large percentage of women took nutritional supplements during the confinement period (95.0 % of women in South Korea and 74.7% of those in China; *p* < 0.001).

Regarding postpartum exercises, Chinese mothers (97.7%) were more likely to engage in at least one type of postpartum exercise compared with Korean women (75.1%). Yoga was popular among both groups (39.8% Korean/71.0% Chinese). Moreover, Chinese mothers (19.9%) were more likely to experience better sleep quality compared with Korean mothers (7.2%). Similar percentages among Korean and Chinese mothers (59 and 57%, respectively) experienced difficulties that were reported as “waking up one-to-two times” or “sleep interruption” during each sleeping cycle.

### Nutrition Education

Results related to nutrition knowledge and relevant education programs are described in Table 2. Nutritional knowledge was assessed with 15 items, and dietary habits and dietary attitude were measured with 10 items each, using a five-point Likert scale. The mean nutrition knowledge scores were 12.36 and 10.61 in the Korean and Chinese groups, respectively. Korean mothers showed a better understanding of nutrition knowledge than Chinese mothers (*p* < 0.001). Moreover, compared with Chinese mothers, Korean mothers were more confident in areas related to nutritional problems, based on the scores for perceived nutrition knowledge but without giving rise to a significant difference. Furthermore, this study indicated that the bulk of participants from both countries requested nutrition education, particularly Korean women, who expressed a stronger desire for such educational programs (*t* = 4.85, *p* < 0.001). Mothers were also asked to recall the experience of participating in educational programs and their perception or willingness to learn through future programs. During the preceding year, 28.5% of Korean and 80.1% of Chinese women reported having participated in a nutrition education program (*p* < 0.001). The data revealed that more Chinese women had the opportunity to receive nutrition information. Similar participant percentages had paid for online classes (6.8% Korean/9% Chinese). The key concern among Korean women related to breastfeeding; among Chinese women, this concerned the cooking method (*p* < 0.001). According to the results, 42.1% of Korean and 40.7% of Chinese mothers chose practice lessons as their favorite type of education approach (*p* < 0.001).

### Satisfaction With a Confined Life

The results for life satisfaction and support during the confinement period are shown in Table 3. Overall, the overall life satisfaction score of Korean mothers was higher compared with Chinese mothers, and the difference was statistically significant (*t* = 6.68, *p* < 0.001). In meal satisfaction part, 0.9% of Korean women choose somewhat dissatisfied, compare with 2.3% of Chinese women. Most people selected somewhat satisfied (42.1% of Korean and 41.2% Chinese), but the very satisfactory proportions, Korean is twice as much as Chinese women, 43.9 and 21.3% respectively. In terms of their husbands' support, the satisfaction of Korean pregnant women is also higher than that of Chinese pregnant women. 46.2 and 36.7% of Korean women choose relatively satisfied and very satisfied respectively. Only 16.3% feel generally satisfied with the support provided by their husbands. Compared with Chinese pregnant women, 22.2% and 13.6% choose relatively satisfied and very satisfied, and most people (61.1%) choose generally.

**Table 3:** Comparison of life satisfaction among Korean and Chinese postpartum women.

**Classification**	**Korea**	**China**	**Total**	**t**	***p***
	**(*n =* 221)**	**(*n =* 221)**	**(*n =* 442)**		
	**M ±SD^†^**	**M ±SD**	**M ±SD**		
Overall satisfaction with the current quality of life	4.16 ± 0.79	3.66 ± 0.81	3.91 ± 0.84	6.68^***^	<0.001
Meal satisfaction during postpartum period	4.29 ± 0.81	3.81 ± 0.65	4.05 ± 0.77	6.83^***^	<0.001
Satisfaction with the husband's support	4.19 ± 0.98	3.46 ± 0.93	3.82 ± 1.02	8.00^***^	<0.001

† *M ± SD represents mean ± standard deviation*.

*** *p < 0.001*.

*Scored by a 5-point Likert scale (1: not satisfied to 5: satisfied)*.

### Postpartum Depression and Childcare Stress

Korean women had significantly higher PPD scores compared with Chinese women (*t* = 2.13, *p* < 0.05). The overall score for Korean mothers was 9.10 (SD ± 5.2), and Chinese mothers' score was 8.20 (SD ± 4.0). The findings revealed that the range of the score for a single question in the Korean group was from 0.46 (SD ± 0.73) to 1.58 (SD ± 0.90). Among the 15 items, 12 received a higher score for CS from Korean compared with Chinese women. The total average scores for CS were 2.81 and 2.73 among Korean and Chinese mothers, respectively, with no significant difference between the two groups. Korean mothers reflected a relatively higher score compared with Chinese mothers (Table 4).

**Table 4:** Comparison of postpartum depression score and childcare stress score among Korean and Chinese postpartum women.

**Classification**	**Korea**	**China**	**Total**	**t**	***p***
	**(*n =* 221)**	**(*n =* 221)**	**(*n =* 442)**		
	**M ±SD**	**M ±SD**	**M ±SD**		
	M ± SD	M ± SD	M ± SD		
Total postpartum depression score	9.1 ± 5.2	8.2 ± 4.0	8.7 ± 4.7	2.13^*^	0.034
Total childcare stress score	2.81 ± 0.68	2.73 ± 0.40	2.77 ± 0.56	1.48	0.139

* *p < 0.05*.

### Influence Factors With Postpartum Problems

Table 5 shows that life satisfaction was significantly and negatively correlated with PPD among Korean and Chinese women (r = −0.379, *p* < 0.001, and r = −0.157, *p* < 0.05, respectively). Conversely, a significantly negative correlation between CS and life satisfaction was observed among the women of both countries (*p* < 0.001). Moreover, PPD and CS were proportional to one another between the Korean and Chinese groups (r = 0.481, and r = 0.184, respectively; both *p* < 0.001).

**Table 5:** Correlation coefficients between childcare stress, postpartum depression, life satisfaction and satisfaction with husband support by country.

**Classification**		**CS**	**EPDS**
Korea	LS^†^	−0.339^***^	−0.379^***^
China		−0.278^***^	−0.157^*^
Korea	SHS	−0.126	−0.112
China		−0.256^***^	−0.166^*^
Korea	EPDS	0.481^***^	/
China		0.184^**^	/

*LS, Life satisfaction; SHS, Satisfaction with the husband support; EPDS, Edinburgh Postnatal Depression Scale; CS, Childcare stress*.

† *Response options: Satisfaction of current life (1: not satisfied to 5:satisfied)*.

* *p < 0.05*,

** *p < 0.01*,

*** *p < 0.001*.

## Discussion

This study investigated post-birth confinement, diet, behaviors, PPD, CS, life satisfaction, and the nutrition education of Korean and Chinese postpartum women and compared all the results between the two groups.

### Traditional Confinement Opinions

Most mothers in South Korea and China followed confinement practices. In the two countries, many similar postpartum practices were prevalent, such as a traditional postpartum diet (22), lying down habits (23), and actions to improve maternal hygiene and heighten the overall quality of life (24, 25). The mothers from these two groups nonetheless differed in terms of specific confinement diets and practices. For example, based on traditional Korean dietary habits, seaweed soup was served to the mother daily after childbirth. People in South Korea follow the notion of “*Saam* (three)-chil (seven)-il (days)”, and the first 3 weeks after delivery are strongly emphasized as part of Korean postpartum care. According to Korean custom, seaweed soup is considered to be part of a uterine involution diet and nutritious food that includes ample calcium, fiber, and iron, which are important in the diet of postpartum women. However, the dietary iodine intake of lactating women was excessively high among South Korean women (26). In contrast, according to traditional Chinese confinement recipes, food that is easy to digest (noodles and porridge) was selected as the first meal post-childbirth. For the first 6 weeks after childbirth, the intake of food was balanced between “hot” and “cold” meals to prevent future maternal diseases. Some Chinese people may also adopt traditional diets such as liquid herbal extracts after childbirth for uterine involution (27).

In terms of behavior, Chinese mothers preferred spending more time at home; however, their sense of requiring exercise was much stronger than that of Korean women during the first 2 months after childbirth. Many studies have shown that the confinement of physical activity patterns contributed to maternal health conditions (11, 28). By focusing on the item “specific frequency of exercise,” while most postpartum women engaged in the confinement exercise described, researchers observed differences in PPD scores, based on the frequency of physical activity. Those who exercised more than three times a week had the lowest PPD score compared with the “once a week” and “no exercise” groups (F = 4.25, *p* < 0.05). Hence, exercising may help mitigate PPD. Contrastingly, exercise status was not clearly linked to PPD, although it appeared to vary inversely with depression in the total study population. In the Korean group, a negative relationship was observed between the desire to exercise and CS (r = −0.148, p < 0.05).

We also explored the sleeping status of postpartum women. The study found that women who slept between 6 and 8 h a day had higher PPD and CS scores than those who slept more than 8 and <6 h (F = 4.21, *p* < 0.05). Although this difference was small, sleeping likely reduces depression and stress. Low total sleeping time was negatively correlated with depression (29).

During the postpartum period, 84.8% of 442 new mothers were found to be nutritional supplements users (95% of Korean and 74.4% of Chinese mothers). Although maternal supplementation appeared to be more common among Chinese mothers at the time of study than in the past, knowledge gaps related to nutrition knowledge between Korean and Chinese women remain significant.

Compared with Chinese mothers, the use of nutritional supplements was more popular among Korean mothers, perhaps due to their stronger emphasis on nutrition. Studies have reviewed the factors associated with the use of nutritional supplements and showed that mothers with a higher education level were more likely to use maternal supplements after giving birth (30). Iron was the most popular supplement among new Korean mothers (29.2%), while 8.1% of Chinese mothers took a daily iron supplement. A higher percentage of supplement use correlated with a stronger focus on the provisioning of professional information. One study noted that mothers failed to take nutritional supplements on time, exposing a lack of knowledge concerning scientific guidelines and, accordingly, a lack of accurate nutrition knowledge (31). Although new mothers in both countries reflected a positive attitude toward and a strong desire for acquiring nutrition knowledge, accurate and sufficient nutrition education service should be improved in the future.

The nutrition education program investigated in the present study indicated the most popular form of education as being practice courses. Moreover, the researchers also analyzed and compared the demands of nutrition education courses between primipara and multipara women.

The primary concern among primipara women concerned breastfeeding (35.3%), whereas, among multipara women, the focus was on improving their cooking skills (27%). The results of the current study showed that, based on CS results, primipara women were significantly more depressed than multipara women (F = 4.65, p < 0.001). Primipara mothers had a stronger need for infant care support and guidance concerning baby-feeding techniques. Therefore, identifying their needs at this stage may result in the better health of both mothers and their infants. Depending on the new mothers' cultural backgrounds and individual characteristics, education methods and content may differ between the two investigated groups.

### Postpartum Depression and Childcare Stress

Korean mothers had higher EPDS and CS scores than Chinese mothers, suggesting that this population was more likely to develop PPD symptoms and childcare-related stress. This result is in agreement with a study conducted by Qobadi (32). Childcare events have consistently been identified as risk factors for PPD. In a meta-analysis, risk factors were summarized and cause-and-effect relationships were indicated between CS, life stress, social support, infant temperament, and PPD (33). In the present research, Pearson correlation analysis showed a positive relationship between spousal support during the postpartum period and psychological health among Chinese women (r = −0.252, p < 0.001). Thus, higher levels of spousal support led to lower levels of CS and PPD among postpartum women. Furthermore, a significantly negative correlation was observed between meal satisfaction, CS, and PPD among Chinese mothers (p < 0.001). Accordingly, we can interpret that, concerning life satisfaction due to positive conditions related to a woman's spouse cooking meals, and providing general care and support in the postpartum period, women managed stress better. Another study identified women's childbirth experience and PPD as factors that influenced their quality of life after childbirth (34).

The strengths of the current study include its high response rate and comparison between South Korean and Chinese mothers. To our knowledge, this is one of the first studies to compare PPD, CS, and life satisfaction, and their related factors among Korean and Chinese postpartum women, thereby indicating cultural differences during the traditional confinement period. The study does, however, include some limitations. Existing research has shown that homemakers experienced higher levels of PPD compared with employed women (35). Furthermore, the specific family type may be another factor that affected PPD and CS scores. Munaf et al. indicated that women from joint families were significantly less depressed than those from nuclear families (36). However, we could not analyze the potential influences of these aspects on the scores of PPD and CS. In addition, there are significant differences between Korean mothers and Chinese mothers in terms of family income, occupation, education level, etc. These confounding factors may have contributed to the difference in the psychological health status among Korean and Chinese postpartum women. We will take these confounding factors into account in future research, and then conduct a more in-depth and comprehensive analysis.

## Conclusion

The postpartum period is a time of transition and social celebration for new mothers and their families that signals a change in social responsibilities. To promote the health of postpartum women in South Korea and China, culturally tailored diet and exercise guidance are crucial. Customizing educational content and methods according to the needs of pregnant women will be conducive to the development and acceptance of such education and can improve its overall effect. In addition, reducing PPD and CS, improving life satisfaction, and enhancing the support provided by spouses are crucial approaches for improving the overall health of new mothers.

## Data Availability Statement

The original contributions presented in the study are included in the article/supplementary material, further inquiries can be directed to the corresponding author.

## Ethics Statement

This study was conducted in accordance with the declaration of Helsinki and approved by the Institutional Review Board of Myongji University (No. MJU2018-10-002-02). The patients/participants provided their written informed consent to participate in this study.

## Author Contributions

JL and KS: conception and design of the research. SK: acquisition of data. HP: analysis and interpretation of the data. YL and HL: statistical analysis. KS: obtaining financing and critical revision of the manuscript for intellectual content. JL and HG: writing of the manuscript. All authors read and approved the final draft.

## Conflict of Interest

The authors declare that the research was conducted in the absence of any commercial or financial relationships that could be construed as a potential conflict of interest.

## Publisher's Note

All claims expressed in this article are solely those of the authors and do not necessarily represent those of their affiliated organizations, or those of the publisher, the editors and the reviewers. Any product that may be evaluated in this article, or claim that may be made by its manufacturer, is not guaranteed or endorsed by the publisher.
